# First Molecular Detection of *Bartonella bovis* and *Bartonella schoenbuchensis* in European Bison (*Bison bonasus*)

**DOI:** 10.3390/ani13010121

**Published:** 2022-12-28

**Authors:** Algimantas Paulauskas, Irma Ražanskė, Indrė Lipatova, Loreta Griciuvienė, Asta Aleksandravičienė, Artūras Kibiša, Dalia Černevičienė, Jana Radzijevskaja

**Affiliations:** Faculty of Natural Sciences, Vytautas Magnus University, K. Donelaičio 58, LT-44248 Kaunas, Lithuania

**Keywords:** *Bartonella bovis*, *Bartonella schoenbuchensis*, *Bison bonsus*, Lithuania

## Abstract

**Simple Summary:**

The European bison is the largest ruminant in Europe. The population of European bison in Lithuania is increasing and several free-ranging herds exist. Owing to their protected status, studies of vector-borne pathogens in European bison are still lacking. By analyzing European bison and ticks collected from them in Lithuania, we checked for the presence of *Bartonella* spp. and found a low frequency (7.9%) of positive animals. This study showed that European bison may be infected by at least two *Bartonella* species, namely *B. bovis* and *B. schoenbuchensis*. Our results demonstrated that further research is needed to determine the distribution of *Bartonella* species in wild and domestic ruminants, and it is important to identify the transmission route.

**Abstract:**

*Bartonella* bacteria infect the erythrocytes and endothelial cells of mammalians. The spread of the *Bartonella* infection occurs mainly via bloodsucking arthropod vectors. Studies on *Bartonella* infection in European bison, the largest wild ruminant in Europe, are lacking. They are needed to clarify their role in the maintenance and transmission of *Bartonella* spp. The aim of this study was to investigate the presence of the *Bartonella* pathogen in European bison and their ticks in Lithuania. A total of 38 spleen samples from bison and 258 ticks belonging to the *Ixodes ricinus* and *Dermacentor reticulatus* species were examined. The bison and tick samples were subjected to *ssrA*, 16S–23S rRNA ITS, *gltA*, and *rpoB* partial gene fragment amplification using various variants of PCR. *Bartonella* DNA was detected in 7.9% of the tissue samples of European bison. All tick samples were negative for *Bartonella* spp. The phylogenetic analysis of 16S–23S rRNA ITS, *gltA*, and *rpoB* partial gene fragment revealed that European bison were infected by *B. bovis* (2.6%) and *B. schoenbuchensis* (5.3%). This is the first report addressing the occurrence of *B. bovis* and *B. schoenbuchensis* in European bison in Europe.

## 1. Introduction

*Bartonella* species are Gram-negative bacteria that infect mammalian erythrocytes and endothelial cells. They are transmitted via direct contact (scratches or bites by infected animals) and by bloodsucking arthropod vectors such as fleas, lice, ticks, sand flies, and deer keds [1,2]. Domestic and wild ruminants can be hosts for *Bartonella bovis*, *Bartonella capreoli*, *Bartonella chomelii*, *Bartonella schoenbuchensis*, and *Bartonella melophagi* [3,4,5,6]. In addition, *Bartonella henselae* and ‘*Candidatus* Bartonella davousti’ have been detected in domestic cattle [7,8]. *Bartonella* species detected in ruminants are rarely implicated in animal diseases. To date, only *B. bovis* has been associated with bovine endocarditis [4,6,9].

In Lithuania, various species of *Bartonella* have been detected in domestic and wild animals and their ectoparasites. *Bartonella grahamii, B. tailorii, B. rochalimae, B. tribocorum, B. coopersplainsensis, B. doshiae,* and *B. washoensis* have been identified in several species of small rodents and in the fleas, ticks or mites collected from them [10,11,12,13]. Furthermore, *B. henselae, B. clarridgeiae* and the *Bartonella* sp., closely related to *B. schoenbuchensis* have been detected in domestic cats and their fleas [14]. However, there is no data on the presence of *Bartonella* pathogens in domestic or wild ruminants in Lithuania.

The European bison (*Bison bonasus*), the largest ruminant in Europe, is known for its status as an animal under species protection. Recent studies have demonstrated that bison may play a role as a natural reservoir of *Anaplasma phagocytophilum, Babesia divergens,* and *Babesia venatorum* [15,16]. However, there is no information about *Bartonella* infection in European bison in Europe. In the present study, we aimed to investigate the presence of the *Bartonella* pathogen in European bison and their ticks (*Ixodes ricinus* and *Dermacentor reticulatus*) in Lithuania using real-time PCR targeting the *ssrA* gene, and to characterize *Bartonella* strains by the PCR and sequence analysis of the 16S–23S rRNA ITS (the 16S–23S rRNA intergenic species region), *gltA* (citrate synthase gene) and *rpoB* (RNA polymerase β-subunit) genes.

## 2. Materials and Methods

### 2.1. Sample Collecting 

A total of 38 spleen samples of European bison (21 males and 17 females) were collected in the period 2019 to 2022 in Central and Northern Lithuania (Kėdainiai and Panevėžys regions). Bison samples were collected in all seasons (*n* = 4 spring, *n* = 1 summer, *n* = 11 autumn, and *n* = 22 winter). Due to the status of animals under species protection, the tissue samples of European bison were taken from animals found dead in the field (*n* = 6), accidentally killed on roads (*n* = 10), and eliminated from nature under a protection plan due to diseases or genetic disorders (*n* = 22). The bison sampling was conducted with permission from the Environmental Protection Department under the Ministry of Environment (permit No. AAA 2019-04-01 use protected species No. 12, in accordance with the protection plan for *Bison bonasus* L.). A total of 258 ticks were collected from 6 animals that had died due to unknown causes. Ticks were placed in 1.5 mL tubes with 70% ethanol and kept at 4 °C until investigation. Tick species were identified based on morphological criteria [17]. Two species of ticks were identified: *Ixodes ricinus* (40 male, 88 female, and 1 nymph) and *Dermacentor reticulatus* (95 male, 34 female). A total of 116 ticks were engorged (85 *I. ricinus* and 31 *D. reticulatus*) and 143 non-engorged (45 *I. ricinus* and 98 *D. reticulatus*).

### 2.2. DNA Extraction and PCR Amplification

Genomic DNA was extracted from bison spleens and engorged ticks using a Genomic DNA Purification Kit (Thermo Fisher Scientific, Vilnius, Lithuania) according to the manufacturer’s recommendations. DNA from non-engorged ticks was extracted using 2.5% ammonium hydroxide [18].

Screening for the presence of *Bartonella* DNA (124 bp product of *ssrA* gene) was conducted by using TaqMan real-time PCR with ssrA-F1 and ssrA-R1 primers and a ssrA-P1 probe, as previously described [11]. *Bartonella*-positive samples in real-time PCR were further analyzed using nested PCR assays that amplify partial sequences of the 16S–23S rRNA ITS region (external primers WITS-F and WITS-R; internal primers Bh311–332F and ITS-R) and conventional PCR assays of *gltA* (primers BhCS.781p and BhCS.1137n) and *rpoB* (primers 1400 F and 2300 R) genes [19,20,21,22] (Table S1). Positive (DNA of *Bartonella*-infected rodents, confirmed by sequencing) and negative (sterile, double-distilled water) controls were included in each PCR run. PCR products were identified by electrophoresis on 1.5 % agarose gel.

### 2.3. DNA Sequencing and Sequence Analysis 

PCR products of all *Bartonella*-positive samples were extracted from agarose gel and purified using the GeneJET™ Gel Extraction Kit (Thermo Fisher Scientific, Vilnius, Lithuania). The obtained partial 16S–23S rRNA ITS region, *gltA,* and *rpoB* gene sequences were analyzed using the MegaX software package [23] and were aligned with the previously published sequences in GenBank using BLASTn. The phylogenetic trees were constructed using the maximum-likelihood method and Tamura-Nei model.

Partial 16S–23S rRNA ITS region, *gltA,* and *rpoB* gene sequences for representative samples obtained in this study were submitted to the GenBank database under accession numbers OP888096 for the ITS region, OP894362–OP894364 for the *gltA* gene, and OP894359–OP894361 for the *rpoB* gene.

## 3. Results

### 3.1. Frequency of Bartonella Infection 

*Bartonella* DNA was detected in three out of the thirty-eight (7.9%) tissue samples of European bison. Positive results were obtained in each animal when applying all three PCR techniques (real-time PCR, and conventional and nested PCR). The *Bartonella* infection was detected in samples collected during winter. All ticks (*n* = 258) collected from the six European bison specimens not infected with *Bartonella* were negative for *Bartonella* spp. All positive samples were subjected to sequence analysis. 

### 3.2. 16. S–23S rRNA ITS Region

**Figure 1:**
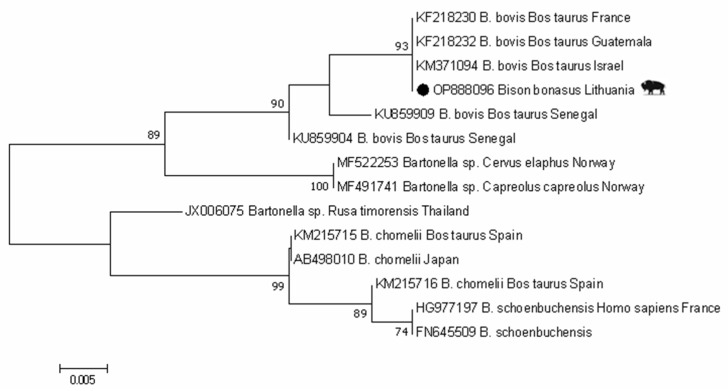
Phylogenetic tree of *Bartonella* spp. using 16S–23S rRNA ITS region sequences constructed by Maximum Likelihood method and Tamura–3 parameter model. Numbers on the tree indicate bootstrap support (values < 50% not shown). The identification source (host) and country are given after the accession numbers. *Bartonella* sample in this study isolated from European bison is marked with ●.

Only one sample of European bison demonstrated the partial 16S–23S rRNA ITS fragment. The obtained sequence (GenBank: OP888096) was 100% identical to *B. bovis* sequences isolated from domestic cattle from France, Guatemala, and Israel (GenBank: KF218230, KF218232, KM371094) (Figure 1).

### 3.3. gltA Gene

**Figure 2:**
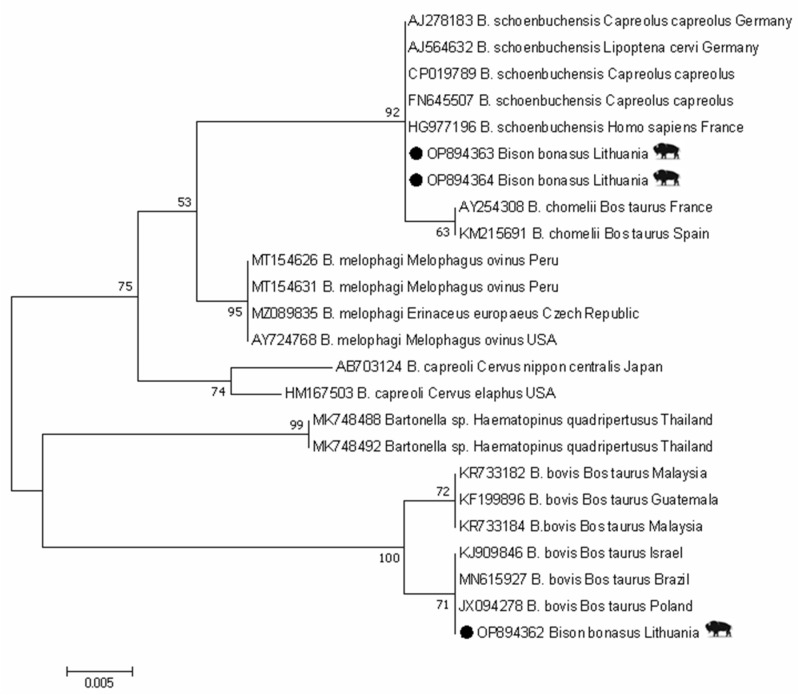
Phylogenetic tree of *Bartonella* spp. using *gltA* gene sequences constructed by Maximum Likelihood method and Tamura–3 parameter model. Numbers on the tree indicate bootstrap support (values < 50% not shown). The identification source (host) and country are given after the accession numbers. *Bartonella* sample in this study isolated from European bison is marked with ●.

In total, three *gltA* partial gene fragments were obtained from three European bison samples. The sequence analysis revealed that the *Bartonella* strain (GenBank: OP894362) derived from one individual European bison clustered with *B. bovis* isolated from cattle from various countries (GenBank: KJ909846, MN615927, JX094278) with an identity score 100%. The other two sequences (GenBank: OP894363, OP894364) demonstrated a 100% similarity with *B. schoenbuchensis* isolated from roe deer (GenBank: AJ278183, CP019789, FN645507), deer ked from Germany (GenBank: AJ564632), and a human sample from France (GenBank: HG977196) (Figure 2).

### 3.4. rpoB Gene

**Figure 3:**
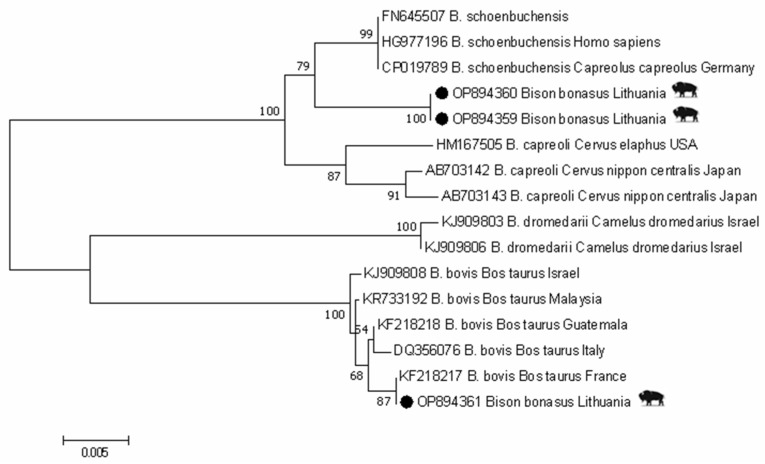
Phylogenetic tree of *Bartonella* spp. using *rpoB* gene sequences constructed by Maximum Likelihood method and Tamura–3 parameter model. Numbers on the tree indicate bootstrap support (values < 50% not shown). The identification source (host) and country are given after the accession numbers. *Bartonella* sample in this study isolated from European bison is marked with ●.

The *rpoB* gene sequence (GenBank: OP894361) clustered with other *B. bovis* sequences. The highest similarity (99.9%) was shared with *B. bovis*, previously isolated from cattle from France (GenBank: KF218217). Meanwhile, two sequences derived from two bison specimens (GenBank: OP894659, OP894360) were 99.1% similar to the *B. schoenbuchensis* sequences isolated from roe deer from Germany (GenBank: CP019789) and human (GenBank: HG977196), differing at six nucleotide positions (Figure 3). Additionally, these sequences shared a 98.5% similarity with *B. capreoli* isolated from red deer from USA (GenBank: HM167505), differing at fifteen nucleotide positions.

## 4. Discussion

This study reports the first molecular detection of *B. bovis* and *B. schoenbuchensis* in European bison in Europe. Furthermore, we tested *I. ricinus* and *D. reticulatus* ticks collected from bison and did not detect the presence of *Bartonella* species. The role of ticks in the transmission of *Bartonella* is not fully understood [4,6]. Other vectors such as deer ked and other fly species may be involved in the transmission of *Bartonella* spp. in ruminants [6,24].

*Bartonella bovis* is mainly detected in cattle. Infection with *B. bovis* is usually asymptomatic, but it is known that it can be associated with bovine endocarditis [4,9]. The prevalence of *B. bovis* in domestic cattle ranges from 7% to 36% in Europe and from 5% to 90% in other continents [4,6,25,26,27]. Furthermore, *B. bovis* infections also have been reported in wild ruminants such as moose, red deer, and roe deer [28,29]. A recent study reported that *B. bovis* DNA has been identified in 3.2% of ticks collected from cattle [6]. Meanwhile, in our study, *Bartonella* infection in ticks has not been detected. This study presents a low frequency of *B. bovis* (2.6%) in European bison in Lithuania. A previous study conducted in Poland has shown that 6.8% of cattle (*Bos taurus*), a closely related species to European bison, had asymptomatic *B. bovis* infection [26]. In order to estimate the prevalence of *Bartonella* in Lithuanian ruminants, an epidemiological study with a larger sample size is needed.

The present study revealed that two individuals of European bison (5.3%) were infected with *B. schoenbuchensis*. This result is not unexpected because previous studies reported cases of *B. schoenbuchensis* in domestic and wild ruminants from Georgia, Poland, France, and Norway [4,28,30,31]. Moreover, this species was determined in the deer keds collected from wild ruminants [31,32]. According to other studies, *Bartonella* spp. highly similar to *B. capreoli*, *B. chomelii*, and *B. schoenbuchensis* had been identified in deer ked distribution areas [29,31,33]. That indicates a potential role of deer ked for the transmission of *Bartonella* species. Furthermore, outside the deer ked distribution area, other vectors such as ticks or culicoides biting midges could also be involved in *Bartonella* transmission [29,34]. European bison are gregarious animals that live and travel in herds. Such a lifestyle provides a possibility for blood-sucking vectors to have a greater effect on the transmission of the diseases [35].

## 5. Conclusions

The results of this study suggest that European bison may be infected by at least two *Bartonella* species, namely *B. bovis* and *B. schoenbuchensis*. Further research is needed to determine the vectors of transmission of *Bartonella* species among bison and other ruminant species.

## Data Availability

The data presented in this study are available within the article.

## Supplementary Materials

The following supporting information can be downloaded at: https://www.mdpi.com/article/10.3390/ani13010121/s1, Table S1: Detailed PCR protocols for *Bartonella* spp. detection.
