# Supplementary material for: First Molecular Detection of Bartonella bovis and Bartonella schoenbuchensis in European Bison (Bison bonasus)
Source: Animals (Basel). 2022 Dec 28;13(1):121. doi: 10.3390/ani13010121 (PMC9817727; doi:10.3390/ani13010121)
Supplement: Supplementary file 1 [file animals-13-00121-s001.zip › animals-2093940-supplementary.pdf]

animals

MDPI

Article

First Molecular Detection of *Bartonella Bovis* and *Bartonella Schoenbuchensis* in European Bison (*Bison Bonasus*)

Algimantas Paulauskas, Irma Ražanskė, Indrė Lipatova, Loreta Griciuvienė, Asta Aleksandravičienė, Artūras Kibiša, Dalia Černevičienė, and Jana Radzijevskaja

Table S1. Detailed PCR protocols for *Bartonella* spp. detection.

Target	1 step PCR	2 step PCR	1st PCR amplification steps	2nd PCR amplification steps
ssrA[a]	1× SensiMix™ II Probe<br>0.2 µM ssrA-F1 Primer<br>0.2 µM ssrA-R1 Primer<br>0.1 µM ssrA-P1 Probe<br>100 ng DNA		95 °C – 10 min<br>95°C – 20 s<br>50 × 50 °C – 1 min<br>72 °C – 10 s	
gltA[b]	1× DreamTaq Green PCR MM<br>0.4 µM BhCS.781p primer<br>0.4 µM BhCS.1137n primer<br>200 ng DNA		95 °C – 5 min<br>95 °C – 1 min<br>40 × 55 °C – 1 min<br>72°C – 1 min<br>72 °C – 5 min	
rpoB[b]	1× DreamTaq Green PCR MM<br>0.8 µM 1400F primer<br>0.8 µM 2300R primer<br>200 ng DNA		96 °C - 2 min<br>94 °C - 50 s<br>45 × 55°C - 50 s<br>72 °C - 1 min<br>72 °C - 5 min	
16 S–23 S rRNA ITS region[c]	1× Taq Buffer with KCl<br>2.5 mM $MgCl_2$<br>0.2 mM dNTP Mix<br>1.5 U Taq DNA Polymerase<br>0.4 µM WITS-F primer<br>0.4 µM WITS-R primer<br>200ng DNA	1× Taq Buffer with KCl<br>2 mM $MgCl_2$<br>0.2 mM dNTP Mix<br>1.5 U Taq DNA Polymerase<br>0.4 µM Bh311-332F primer<br>0.4 µM ITS-R primer<br>1 µl PCR product	96 °C - 2 min<br>94 °C - 50 s<br>40 × 48 °C - 50 s<br>72 °C - 1 min 30 s<br>72 °C - 10 min	96 °C - 2 min<br>94 °C - 45 s<br>45 × 60 °C - 30 s<br>72 °C - 45 s<br>72 °C - 5 min

[a] Real time PCR [b] Conventional PCR [c] Nested PCR
